# Owner-rated hyperactivity/impulsivity is associated with sleep efficiency in family dogs: a non-invasive EEG study

**DOI:** 10.1038/s41598-023-28263-2

**Published:** 2023-01-23

**Authors:** Cecília Carreiro, Vivien Reicher, Anna Kis, Márta Gácsi

**Affiliations:** 1 Doctoral School of Biology, Institute of Biology, ELTE Eötvös Loránd University, Budapest, Hungary; 2 Department of Ethology, Institute of Biology, ELTE Eötvös Loránd University, Budapest, Hungary; 3 MTA-ELTE Comparative Ethology Research Group, Budapest, Hungary; 4 Institute of Cognitive Neuroscience and Psychology, Research Centre for Natural Sciences, Budapest, Hungary

**Keywords:** Circadian rhythms and sleep, ADHD

## Abstract

Subjective sleep disturbances are reported by humans with attention-deficit/hyperactivity disorder (ADHD). However, no consistent objective findings related to sleep disturbances led to the removal of sleep problems from ADHD diagnostic criteria. Dogs have been used as a model for human ADHD with questionnaires validated for this purpose. Also, their sleep physiology can be measured by non-invasive methods similarly to humans. In the current study, we recorded spontaneous sleep EEG in family dogs during a laboratory session. We analyzed the association of sleep macrostructure and deep sleep (NREM) slow-wave activity (SWA) with a validated owner-rated ADHD questionnaire, assessing inattention (IA), hyperactivity/impulsivity (H/I) and total (T) scores. Higher H/I and T were associated with lower sleep efficiency and longer time awake after initial drowsiness and NREM. IA showed no associations with sleep variables. Further, no association was found between ADHD scores and SWA. Our results are in line with human studies in which poor sleep quality reported by ADHD subjects is associated with some objective EEG macrostructural parameters. This suggests that natural variation in dogs’ H/I is useful to gain a deeper insight of ADHD neural mechanisms.

## Introduction

Attention-deficit/hyperactivity disorder (ADHD) is one of the most prevalent psychiatric conditions^1^, being characterized by symptoms of inattention (IA) and/or hyperactivity/impulsivity (H/I)^2^. Patients with ADHD might also show impairments in the academic and social domains^3^. Although sleep disturbances were removed from the diagnostic criteria for ADHD due to the lack of empirical evidence^4,5^, several studies indicated that sleep disturbances are more common in ADHD patients of all ages than in the general population^6–8^. In addition to subjective measures (e.g., parent questionnaire reports^9^), objective sleep parameters (e.g., actigraphy^10^; polysomnography^11^) were also applied to explore the associations between sleep and ADHD.

Regarding electroencephalography (EEG) sleep macrostructure, previous findings indicated that ADHD children had lower sleep efficiency than the control; meanwhile, in sleep parameters of NREM (stage 1, 2 and 3), REM and REM sleep latency, no differences were observed^6^. However, a more recent meta-analysis found significant differences, but only in NREM stage 1; specifically, ADHD children spent more time in stage 1, indicating a lighter sleep compared to controls^12^. Interestingly, no findings were replicated in adults with ADHD^7^. Discrepancies between the meta-analyses might be explained by the fact that Cortese et al.^6^ included studies in which children had primary sleep disorders. In such cases, it is problematic to conclude if sleep disturbances in the ADHD group were actually due to ADHD itself or if the results were biased by the primary sleep problems. Furthermore, most of the studies did not differentiate ADHD subjects into subtypes. In the few studies when individuals are separated by their ADHD subtype diagnosis, sleep disturbances were different depending on symptoms, comorbidities and medications (e.g.^13,14^).

With respect to spectrum power differences, most studies focus on slow wave activity (SWA; EEG power in the 0.75–4.5 Hz band) in NREM sleep, as this parameter has a crucial role in synaptic homeostasis^15^, plasticity^16^ and memory consolidation^17^. Previous studies—using high density EEG—observed higher levels of SWA in NREM sleep in ADHD children in the centro-posterial scalp derivation^18–20^. Yet, another study reported reduced SWA across the scalp in children and adolescents with ADHD compared to controls^11^. A longitudinal sleep study reported no group differences between ADHD and the control, yet the percent of SWA in the first NREM period was lower in the ADHD group^21^. According to a recent meta-analysis, the observed differences might reflect developmental alterations, as spectral differences in ADHD patients seem to be age-related; increased SWA in early childhood and decreased SWA in late childhood/adolescence, with the transition point at around 10 years old^22^. In adults, sleep spectral parameters were less studied. One study found increased SWA in ADHD individuals compared to non-ADHD controls^23^, while another study reported no differences between control and ADHD groups^24^.

Thus, it is clear that even more robust and sophisticated investigations are required to better understand the associations between ADHD and sleep. Incorporating relevant animal models into the investigation offers a promising opportunity to clarify these divergent findings and to expand our knowledge in how ADHD affects sleep. These models can provide insight into specific aspects of disorders, which cannot be gained from research with humans due to practical and ethical reasons^25,26^.

Several studies have demonstrated that the family dog is a promising animal model for human socio-cognitive behaviors as well as their neuro-cognitive background. This includes attachment (behavior^27^; EEG^28^; neuroimaging^29^), voice processing (behavior^30^; ERP^31^; neuroimaging^32^) and learning (behavior^33^; EEG^34,35^). Furthermore, the family dog is increasingly recognized as a model for human neuropsychiatric conditions, such as obsessive–compulsive disorder^36^, autism^37^ and ADHD-like characteristics^38^. Different questionnaires, originally designed to measure human behavior, have been successfully applied to measure dog behavior (personality^39^; impulsivity^40^). The first questionnaire validated for dogs to evaluate ADHD-like traits^41^ was developed on the basis of a human parental questionnaire, the DuPaul ADHD Rating Scale-IV^42^. Its reliability was validated and its factor structure was replicated by an independent study^43^.

Behavioral tests have also been applied to assess ADHD-like traits in dogs. Higher owner-rated H/I scores were associated with intolerance on delayed reward^44^; dogs with higher H/I scores preferred immediate reward even though the food amount was smaller. Likewise, dogs that displayed higher social impulsivity (faster approach towards friendly strangers) had a positive association with dopamine receptor gene polymorphisms, which are known to be related to ADHD^45^. Recent findings on canine self-inhibition using a behavioral Go/No-Go paradigm showed that dogs’ higher H/I scores were associated with a greater number of mistakes due to poorer self-inhibition, and dogs’ higher IA scores were associated with a shorter time to make these mistakes^38^. These findings are in line with previous results on children with ADHD (e.g.^46^). Importantly, none of these questionnaires or the behavioral tests have been dedicated to diagnosing ADHD in dogs. In our study, the questionnaire was also applied to assess characteristics of the ADHD-like spectrum.

In this study, we present the first step to assess associations between ADHD scores and sleep EEG parameters in the dog. The increasing interest in canine sleep research stems from its advantages to investigate the sleep of a domesticated species, which in many respects is comparable to human sleep (for review, see^47^). Due to dogs’ natural cooperativeness, untrained family dogs have already been measured in a number of different non-invasive sleep EEG studies (e.g.^28,48^). We assumed that dogs’ ADHD factors (IA, H/I and total scores based on^41^) would show similar associations with sleep EEG variables to that of humans with ADHD. Specifically, dogs with higher scores on ADHD were expected to sleep less (lower sleep efficiency), spend more time awake after falling asleep (more wakefulness after sleep onset, WASO) and spend more time in superficial sleep (drowsiness: transitional stage between quiet wakefulness and sleep^49^). Regarding SWA (1–4 Hz), we did not formulate a clear hypothesis due to the controversial results reported in the human literature. In the present study, we explored any potential associations between dogs’ ADHD scores and SWA during NREM sleep.

## Method

This study was approved by the Scientific Ethics Committee for Animal Experimentation (Állatkísérleti Tudományos Etikai Tanács) of Budapest, Hungary, by categorizing it as a non-invasive study (number of ethical permission: PE/EA/853-2/2016). The experimental protocols were conducted according to the guidelines of the Declaration of Helsinki and to ARRIVE guidelines, as outlined by the Association for the Study Animal Behaviour (ASAB). Owners participated with their dogs in this study without any monetary compensation and gave written consent.

The location of the sleep measurements was in one of the two fully equipped laboratories for canine EEG measurements, depending on the laboratory availability. One laboratory is located at the Eötvös Loránd University (ELTE) and the other one is located at the Research Centre for Natural Sciences, Institute of Cognitive Neuroscience and Psychology.

A more detailed description of the protocols below can be found in Supplementary Information.

### Subjects

A total of 86 family dogs were recruited from the Family Dog Project (ELTE, Department of Ethology) database. They participated in a sleep EEG session during daytime in the presence of the owners and had an ADHD questionnaire rated by their owners. In this study, we analyzed dogs between 6 months and 14 years of age (M_*age*_ in months ± SD: 70.45 ± 52.10), including 36 males and 50 females of various breeds (54 purebred dogs and 32 mixed breed dogs).

### Attention-deficit/hyperactivity disorder questionnaire

Dogs’ owners were asked to answer the ADHD questionnaire validated for dogs^41^, which assesses individual differences of inattention (IA; 6 items of attentional levels), hyperactivity/impulsivity (H/I; 7 items of motor activity/impulsivity levels) and the combination of total scores (T; 13 items). The questionnaire was developed based on a validated version used for a parent-report rating scale of ADHD and related problems in children (ADHD-RS-IV^42^). Higher scores indicate greater difficulties with IA and H/I. In addition to IA and H/I scores, total (T = IA + H/I) score was also evaluated.

Statements related to attention and activity were presented in the same mixed order. The owners had to choose among the given answers, representing how frequent the statement was true for their dogs on a Likert scale. Initially, the questionnaire was given to the owners in a paper form and, later, it was converted to an online version. During to this transition from the paper to the digital questionnaire, a fault happened related to the answer options and the 4-point Likert scale (N = 34) was changed to a 5-point Likert scale (N = 52). To use all the 86 answers as a unified database, we transformed the scales into a uniform 5-point Likert scale, using normalized scores. The details of the transformations can be found in the Supplementary Information (Fig. S1, Table S1).

### Electroencephalography

Prior the sleep EEG measurements to have dogs’ sleep as standardized as possible, owners were instructed to keep the dogs in a typical routine. This means that dogs slept normally the night before, woke up as usual in the morning and had no extra activity or stress, as variations in dogs’ routine are known to increase sleep pressure and affect sleep variables (e.g.^49^). 

**Figure 1:**
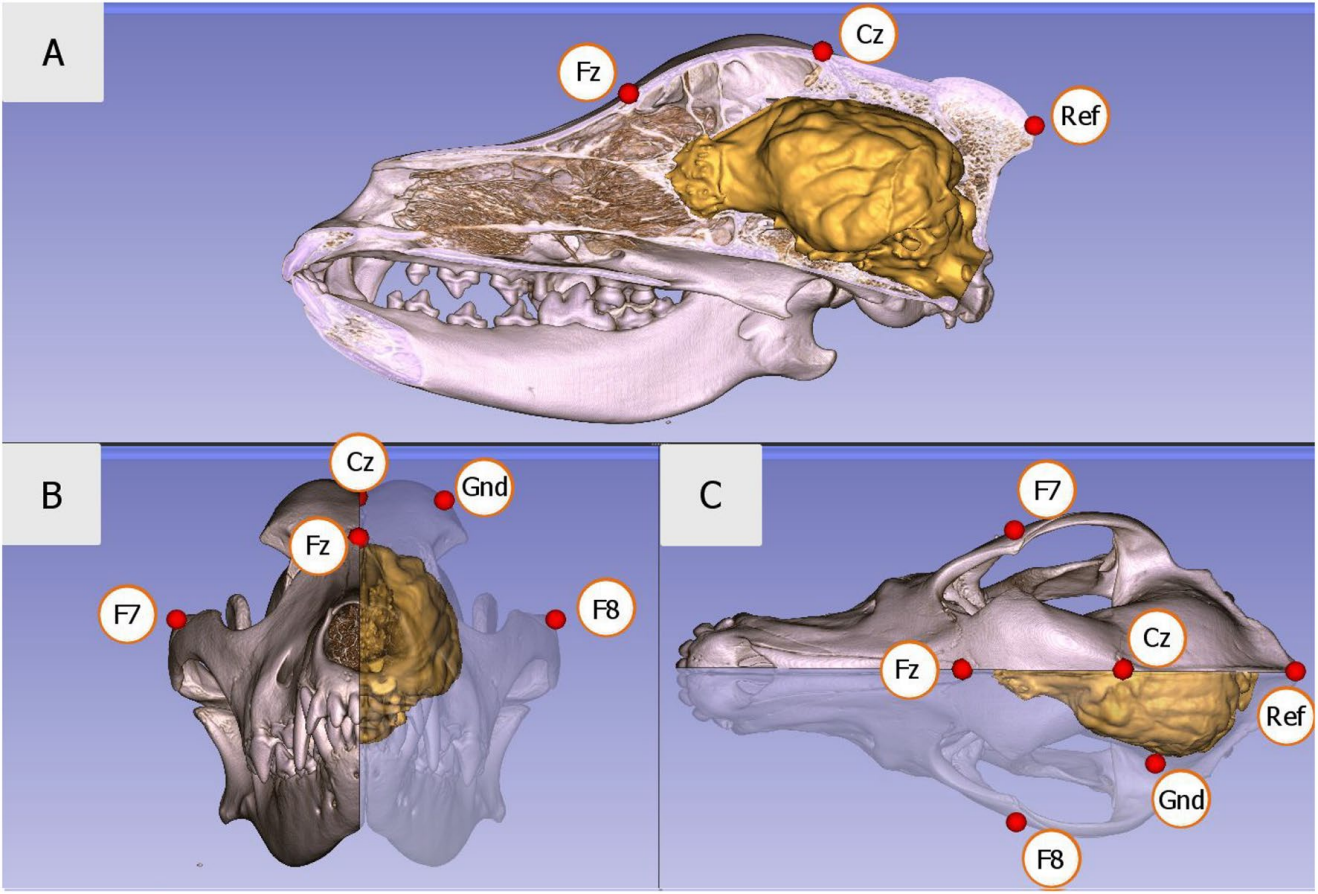
Positions of the electrodes relative to the three-dimensional model and endocranial cast of the skull of a pointer dog: (**A**) lateral, (**B**) anterior and (**C**) superior views, image by Kálmán Czeibert. Placement of the electrodes (Fz–Cz: frontal and central midline; F8–F7: right and left electrodes placed on the zygomatic arch; Ref: reference electrode or G2; Gnd: ground electrode or G1). In this study, the statistical analysis was performed with the Fz data only. All other electrodes were merely used to aid sleep stage scoring.

Dogs were assessed in a non-invasive sleep EEG recording with the minimum duration of 1.5 h and a maximum duration of 3 h during daytime (depending on owners’ availability; 70% started in the afternoon (12–18 h), 25% in the evening (18–20 h) and 5% before noon). The variance in the record duration occurred due to differences in subject compliance. Some dogs after waking up became very active, thus, the recording had to be finished regardless of elapsed time. Other dogs, even if they woke up, continued lying and relaxing next to their owner and/or fell back asleep (thus, recording could be continued for the duration of 3 h). A detailed description of the most recent polysomnographic method and EEG electrode placement (Fig. 1) can be found in Reicher et al.^50^.

We followed the validated polysomnography (PSG) method on dogs^49^. Dogs were measured between 2015 and 2020. During this period, electrode placement and recording methods were improved (for detailed description of the most recent method, see^50^). In the case of 72 dogs, the old setup was used, thus, one active EEG channel (frontal: Fz) and an eye movement channel were recorded. While in the case of 14 dogs, the current setup was used, specifically, four active EEG channels and an eye movement channel were recorded. In this case, Fz and Cz channels were placed over, respectively, the anteroposterior midline of the skull; F7 and F8 channels were placed on the right and left zygomatic arch next to the eyes, all referred to G2. The ground electrode (G1) was attached to the left *musculus temporalis*. Also, an additional channel, labeled EOG, was visualized as the bipolarly referenced F7-F8 electrodes. In all dogs, at least the frontal electrode (Fz) was active, thus, in this study only data from this electrode were used for spectral analyses. See Fig. 1 for electrode placement. Gold-coated Ag/AgCl electrodes were used, secured by Signa Spray Electrode Solution (Parker, United States) and EC2 Grass Electrode Cream (Grass Technologies, United States). Impedance values of the EEG electrodes were kept under 20 kΩ during the recordings.

Recordings were obtained with one of the following technical arrangements:

1. In case of 14 dogs, the signal was collected, amplified and digitized at a sampling rate of 1000 Hz/channel using the 40-channels NuAmps amplifier (© 2018 Compumedics Neuroscan) and DC-recording, later saved in .cnt format with the Scan 4.3 Acquire software (© 2018 Compumedics Neuroscan) then converted to .edf format using MatLab EEG Toolbox.
2. In case of 72 dogs, the signals were collected, pre-filtered, amplified and digitized at a sampling rate of 1024 Hz/channel, using the 25 channel SAM 25R EEG System (Micromed, Mogliano Veneto, Italy), and the System Plus Evolution software with second-order filters at 0.016 Hz (high pass) and 70 Hz (low pass).

To correct for differences in EEG filter characteristics across recording devices, a standard calibration process (dog^51,52^; human^53,54^) was implemented on devices (1) and (2). For more details, see Supplementary Information.

### Data analysis

The sleep EEG assessment followed the protocol described by Reicher et al.^50^. Both sleep macrostructure and spectral data were analyzed. Sleep macrostructure variables were examined in 86 dogs (M_*age*_ in months ± SD: 70.45 ± 52.10) and the spectral variable in 70 dogs (M_*age*_ in months ± SD: 69.91 ± 51.40) as 16 dogs did not reach NREM sleep during their sleep recording.

Sleep recordings were visually scored in accordance with standard criteria^55^ adapted for dogs, previously shown to reliably identify stages of wake, drowsiness, NREM and REM in dogs^49,56^. A program developed by Ferenc Gombos (Fercio’s EEG Plus, 2009–2022) was used to analyze and export data. The recordings were manually scored, artifacts on the EEG channels were excluded and the program provided data of macrostructure and spectrum variables from the different sleep stages.

The macrostructural variables of interest were sleep efficiency, relative time spent awake after the first epoch scored as drowsiness (WASO 1) and after NREM sleep (WASO 2), relative time spent in drowsiness, NREM and REM sleep. Due to the variance of total record time, relative values were used in the analyses for all sleep macrostructure variables to control for any potential biases. Regarding spectral analysis, due to the relevance of SWA in deep sleep for the restorative function of sleep and ADHD (e.g.^21,22^), we analyzed only this frequency range. Average power spectral densities were calculated by a Fast Fourier Transformation (FFT) algorithm applied to the 50% overlapping Hanning-tapered 4-s windows of the EEG signal of the Fz-G2 derivation. Dogs are known to show notable individual-level variation in morphological features regarding head musculature and skull shape and thickness^57^ that might influence the EEG data. To circumvent measurement error that might arise from these differences, absolute power was normalized by computing the relative power spectra of the Fz data for SWA in the frequency range of 1–4 Hz. This means that the absolute spectrum values for the 1–4 Hz frequency range on the Fz channel were divided by the sum of the absolute Fz values for the 1–30 Hz range.

The macrostructure and spectrum variables of interest are summarized in Table 1.

**Table 1:** Variables of interest regarding dogs’ ADHD questionnaire and sleep EEG data. (N)REM: (non)rapid eye movements sleep stage.

Assessment	Variable	Measure
Dog ADHD questionnaire	T score	total sum of all 13 items (1–65)
	IA score	sum of 6 items related to inattention (1–30)
	H/I score	sum of 7 items related to hyperactivity/impulsivity (1–35)
EEG data	Sleep efficiency	time spent sleeping/record duration (%)
	WASO 1	time spent awake after the first epoch scored as drowsiness/record duration (%)
	WASO 2	time spent awake after the first epoch scored as NREM/record duration (%)
	Drowsiness	time spent in drowsiness/record duration (%)
	NREM	time spent in NREM/record duration (%)
	REM	time spent in REM/record duration (%)
	SWA	slow-wave activity relative power spectrum (frequency range: 1–4 Hz)

### Statistical analysis

The statistical analyses were performed in R (version 3.6.3: RCoreTeam, 2014). ADHD variables (IA, H/I and T scores) as well as most sleep parameters (except from WASO 1 and WASO 2) showed non-normal distribution based on Shapiro-Wilkson normality test. Prior further analysis, age-related differences in our variables were also checked with Kendall’s partial rank correlations as age had non-normal distribution. Only NREM and the SWA were affected by age. Thus, in the case of the final analysis of ADHD-related differences in NREM sleep and in SWA, partial correlations were performed, controlling for age. Kendall’s rank correlations were conducted between ADHD-related variables and sleep parameters (sleep efficiency, WASO 1, WASO 2, drowsiness, REM, NREM and SWA). To control for multiple comparisons, Benjamini–Hochberg correction was conducted.

Additionally, since the dogs’ sleep was measured at different hours of the day (i.e. different circadian rhythms) due to practical reasons (availability of the owners), we ran Pearson correlations to check whether the EEG recording start time was related to the ADHD scores used for correlation analyses and to the EEG variables.

## Results

See Table 2 for a summary of statistical results.

**Table 2:** Results of Kendall tau’s B correlations between ADHD and sleep related variables (* statistically significant).

Independent variable	Dependent variable	Kendall tau’s B	*p* value	corr. *p* value	Confidence intervals
Total	Sleep efficiency	−0.197	0.008*	0.031*	−0.342, −0.053
	WASO 1	0.196	0.009*	0.031*	0.051, 0.341
	WASO 2	0.223	0.003*	0.031*	0.070, 0.376
	Drowsiness	0.109	0.146	0.245	−0.039, 0.257
	NREM	−0.065	0.377	0.465	−0.210, 0.099
	REM	−0.118	0.188	0.225	−0.260, 0.023
	SWA	0.055	0.501	0.554	−0.092, 0.236
Inattention	Sleep efficiency	−0.172	0.024	0.062	−0.317, −0.028
	WASO 1	0.164	0.031	0.073	0.017, 0.311
	WASO 2	0.181	0.018	0.053	0.026, 0.336
	Drowsiness	0.109	0.152	0.245	−0.037, 0.256
	NREM	−0.073	0.323	0.424	−0.202, 0.100
	REM	−0.083	0.282	0.394	−0.233, 0.067
	SWA	0.024	0.765	0.765	−0.068, 0.258
Hyperactivity/Impulsivity	Sleep efficiency	−0.208	0.006*	0.031*	−0.352, −0.065
	WASO 1	0.198	0.009*	0.031*	0.056, 0.339
	WASO 2	0.203	0.007*	0.031*	0.052, 0.354
	Drowsiness	0.093	0.221	0.331	−0.055, 0.240
	NREM	−0.051	0.489	0.554	−0.212, 0.108
	REM	−0.133	0.082	0.172	−0.265, −0.002
	SWA	0.042	0.608	0.638	−0.160, 0.194

**Figure 2:**
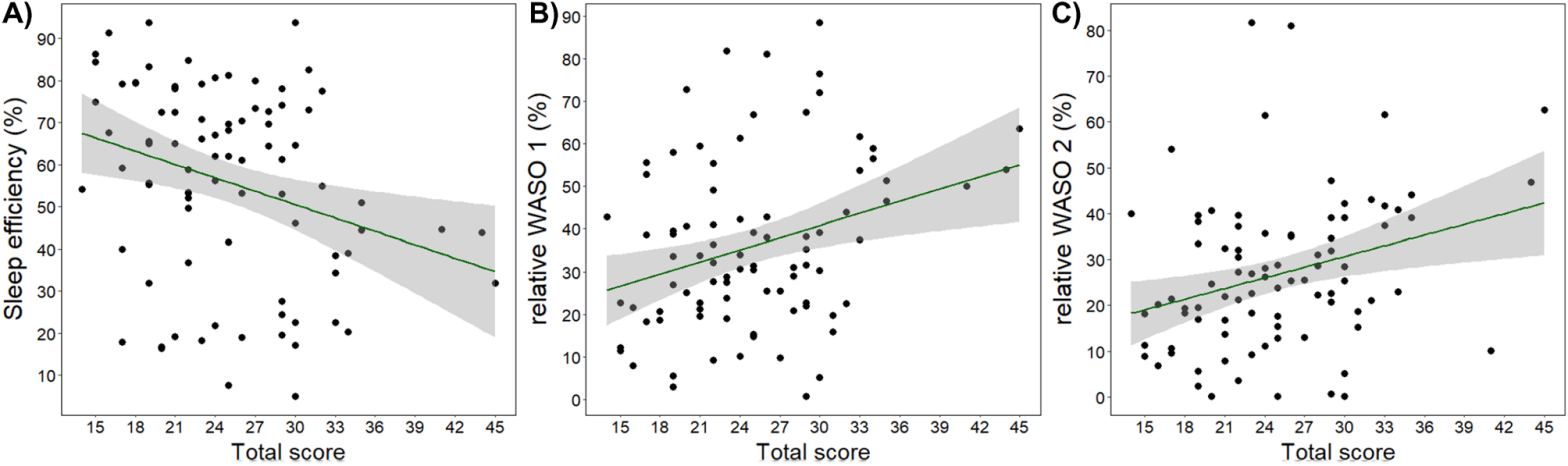
Visualization of Kendall’s rank correlations that showed significant associations between ADHD total score and (**A**) sleep efficiency (*τ*_b_ = −0.197, corr. *p* = 0.031), (**B**) WASO 1 (*τ*_b_ = 0.196, corr. *p* = 0.031) and (**C**) WASO 2 (*τ*_b_ = 0.223, corr. *p* = 0.031) after controlling for multiple correlations (Benjamini-Hochberg). Shaded areas indicate confidence intervals.

There was a negative association between ADHD T scores and sleep efficiency, and a positive association between T scores and WASO 1 and WASO 2 (Fig. 2). Actual power analysis showed that these associations had a medium effect size (respectively, Cohen’s d = 0.573; 0.569; 0.668; provided by G*Power version 3.1.9.7^58^). T scores were not associated with drowsiness, NREM and REM sleep duration, and SWA.

**Figure 3:**
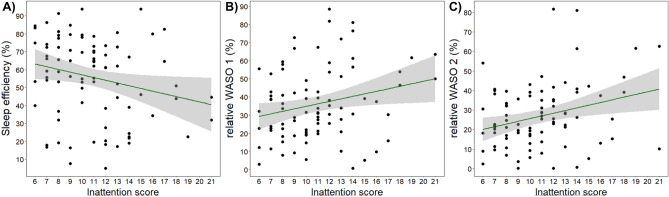
Visualization of Kendall’s rank correlations that showed non-significant tendency between ADHD inattention score and (**A**) sleep efficiency (*τ*_b_ = −0.172, corr. *p* = 0.062), (**B**) WASO 1 (*τ*_b_ = 0.164, corr. *p* = 0.073) and (**C**) WASO 2 (*τ*_b_ = 0.181, corr. *p* = 0.053) after controlling for multiple correlations (Benjamini–Hochberg). Shaded areas indicate confidence intervals.

After controlling for false discovery rate due to multiple comparisons, the associations between IA and the sleep variables were no longer significant (Fig. 3).

**Figure 4:**
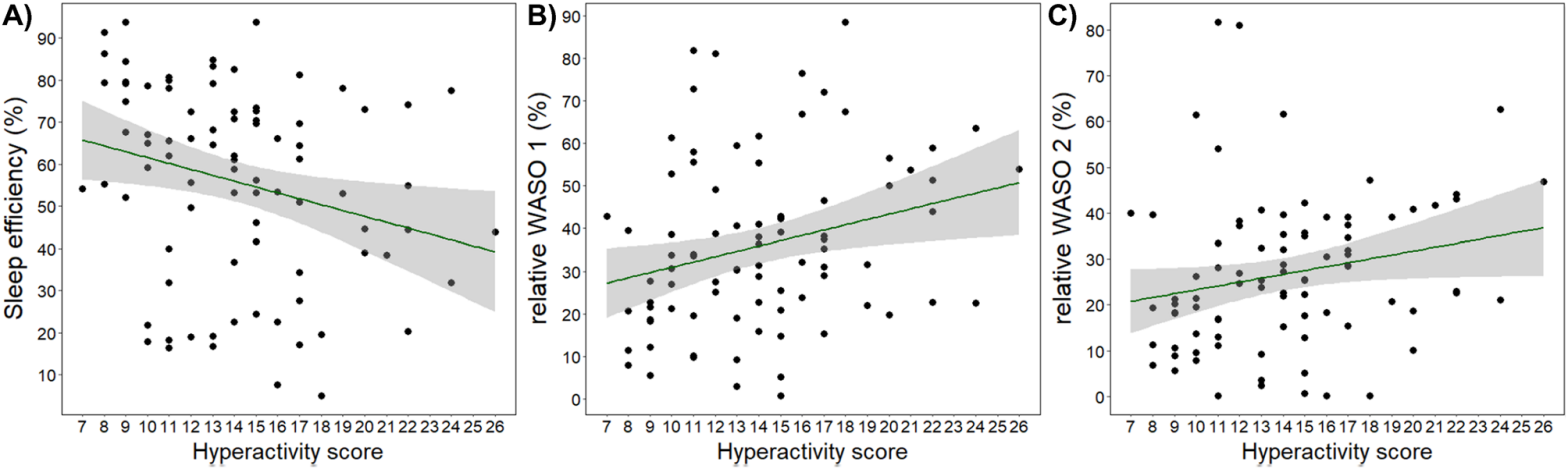
Visualization of Kendall’s rank correlations that showed significant associations between ADHD hyperactivity/impulsivity score and (**A**) sleep efficiency (*τ*_b_ = −0.208, corr. *p* = 0.031), (**B**) WASO 1 (*τ*_b_ = 0.198, corr. *p* = 0.031) and (**C**) WASO 2 (*τ*_b_ = 0.203, corr. *p* = 0.031) after controlling for multiple correlations (Benjamini–Hochberg). Shaded areas indicate confidence intervals.

H/I was negatively associated with sleep efficiency and positively associated with WASO 1 and WASO 2 (Fig. 4). Actual power analysis showed that these associations had a medium effect size (respectively, Cohen’s d = 0.573; 0.569; 0.668; provided by G*Power version 3.1.9.7^58^). Furthermore, H/I was not associated with drowsiness, NREM and REM sleep duration, and SWA.

There was no association between the ADHD scores (T: r = −0.059,* p* = 0.590; IA: r = −0.064, *p* = 0.560; H/I: r = −0.037, *p* = 0.737) and the EEG recording start time, as well as no association between EEG variables (Sleep efficiency: r = 0.053, *p* = 0.624; WASO 1: r = −0.003, *p* = 0.973; WASO 2: r = −0.011, *p* = 0.916) and the EEG recording start time.

## Discussion

We examined whether family dogs’ owner-rated ADHD scores are related to their sleep structure and NREM EEG spectrum. Our results supported our hypothesis regarding sleep efficiency, but did not in case of drowsiness and we did not find associations between ADHD scores and slow wave activity.

Dogs with higher T and H/I scores showed lower sleep efficiency and a similar tendency was detected in case of IA. Similarly, children^6^ and adults^59^ diagnosed with ADHD were reported to have lower sleep efficiency. This parallel holds true despite the fact that the current dog study used the ADHD score as a continuous variable, while previous human studies compared ADHD versus control individuals using a set cut-off. However, according to a more recent PSG-based meta-analysis, no differences in sleep efficiency were detected between control and ADHD groups in either children^12^ or adults^7^. It is noteworthy that most human studies, focusing on the associations between ADHD and sleep, measured individuals with ADHD as a universal group and did not differentiate between subtypes (for review, see^7^). One of the exemptions, somewhat similarly to our findings, found that the H/I subtype had lower sleep efficiency, while IA and combined subtypes had no significant differences in their sleep^60^. Interestingly, one study, based on parental rating, found that children of IA subtype had the fewest reported sleep problems compared to H/I and combined subtypes^13^. Other studies reported different associations; compared to the combined subtype, IA subtype was associated with poorer sleep quality in adults^61^. The diverse results suggest differences according to specific ADHD subtypes (for review, see^62^).

In the current study, the time dogs spent awake after drowsiness (WASO 1) and after NREM sleep (WASO 2) onset also showed correlations with ADHD scores. Higher T and H/I were positively associated with WASO 1 and 2. These results are in line with our findings about sleep efficiency (i.e. if dogs sleep less, they spend more time awake either at the beginning of the recording or after sleep onset). Regarding awakenings and specific ADHD subtypes, fewer data is available in human literature. A study using EEG found that ADHD H/I children had greater sleep fragmentation^60^. Another study analyzing abnormal movements in ADHD children during the night suggested higher sleep fragmentation compared to control^63^, which is in line with increased WASO^64,65^. Most of these studies used different indices to quantify movements in sleep and the data cannot be directly compared, but they all are indicative of more awakenings and sleep fragmentation in ADHD.

We did not find associations between ADHD scores and drowsiness, NREM and REM sleep duration. It is difficult to parallel our results with previous human findings as the human literature shows inconsistent results. Cortese et al.^6^, in a meta-analysis, observed no associations between ADHD and sleep macrostructure variables in children. A more recent meta-analysis found that children with ADHD spent more time in stage 1 NREM, compared to control^12^. In adults, however, a replication study did not find differences between ADHD and control^7^. We have to note that, despite all our efforts to have pre-sleep activity uniform across subjects, there might be still a consistent bias between high versus low ADHD dogs in this respect (e.g. dogs scoring high on the H/I factor are expected to spontaneously engage in more activity during their typical routine). It is known that increased pre-sleep activity affects subsequent sleep structure, causing, e.g., increased sleep efficiency^49,66^. In contrast, our current findings show, in accordance with the human literature on ADHD sleep^6,59^, that dogs with higher T scores (which includes the activity factor) had lower sleep efficiency. Moreover, in our dogs, the different hours the EEG started (i.e. different stages of the circadian rhythms) had no association with the ADHD scores nor with the EEG variables. Thus, it is unlikely that the result reported here would be due to differences in pre-sleep activity since that would predict a difference in the opposite direction. However, it is possible that our results are the sum of effects from differences inherent in the ADHD factors and differences in pre-sleep activity; this would mean that, e.g., the difference in sleep efficiency is actually greater than reported here, but it was partly offset by the dogs’ physical activity. Following the same line of thought, it is possible that other sleep macrostructure differences could have been entirely masked, showing non-significant findings. Disentangling these separate effects is obviously near to impossible, not only in dogs, but also in humans, since interfering with the usual routine of subjects in order to unify pre-sleep activity would inherently result in stress for those implicated and, as a result, it would bias the sleep data yet again. 

With respect to SWA, after controlling for age, we found no associations with ADHD scores. This is contrary to the human literature; ADHD delayed SWA decline during brain maturation seems to be more evident in adolescents (12–16.5 years old^21^). Thus, it has been suggested that SWA dynamics and sleep homeostatic recovery may occur more slowly, specifically, in ADHD adolescents than in other age groups. In our study, although there was a large age variability, we did not separate our subjects in different age groups due to the sparse literature on clear age ranges from puppies to senior dogs (e.g.^51^). Furthermore, in dogs, it is not clear at what age the onset of SWA decrease occurs and what other factors affect it. For instance, in our previous study, we found that SWA was not only associated with age, but with dogs’ weight as well; larger-sized dogs had more SWA than smaller-sized ones (for more details, see^51^). In addition, most recent studies mapped different topographical features in ADHD children, using high-resolution EEG method^18,20^. In dogs’ sleep EEG studies, four EEG channels are applied (e.g.^67,68^) and, in the current research, only the frontal EEG channel was analyzed. Thus, alternations in cortical topography of dog sleep could not be investigated.

Besides these physiological factors, psychosocial factors have also been reported to be involved in the multiple underlying pathways of ADHD^69^. Thus, ADHD has been seen as a socio-emotional dysfunction. These would be important to consider in future canine research as, e.g., the dog-owner social bond is in association with altered SWA dynamics during sleep (alpha-delta anticorrelation^28^).

It is important to emphasize that dogs that participated in this study did not receive the diagnosis of ADHD, thus they represent the typical family dog population and their levels of IA and H/I. This implies a relevant difference from human studies that are always composed of subjects diagnosed for ADHD. A sample focused on dogs with the highest ADHD scores would benefit comparative analyses, approaching to more similar behavioral disorders in human ADHD patients. Also, it is well documented that ADHD symptoms tend to decrease from childhood to adulthood and studies in humans analyze individuals in separated age groups^24,70^. Due to practical reasons, however, the age range in our study was wider than usual human ADHD research. Thus, it would be fruitful to analyze potential differences within and between specific age groups in future studies with dogs.

Our study considered EEG data only from the first time the dogs were assessed in an unfamiliar place during daytime, constituting a different routine during their sleep time compared to sleeping at home in nighttime^66^. In humans, sleep studies are mainly conducted during nighttime. This difference between human nighttime and canine daytime sleep measurements further challenges the comparisons. To handle this difference of daytime sleep, as part of our EEG protocol, owners were instructed to keep the dog on a typical day, e.g., no extra activity carried out or sleep deprivation, which could increase sleep pressure and affect sleep variables as shown in previous sleep studies in dogs^49,66^. However, we did not have information on the exact duration of time spent awake and this specific control (e.g. different length of time dogs are awake before the sleep EEG) would be interesting for further studies on sleep pressure and SWA. When analyzing the effect of other factors such as daytime versus nighttime sleep, pre-sleep activity and location, Bunford et al.^66^ observed that dogs had, e.g., a longer WASO 1 in daytime sleep, indicating that the time of the sleep related to ADHD needs further analysis. However, it is important to highlight that our findings are similar to studies on the effect of ADHD in WASO 1 in humans (e.g.^60^). The findings of pre-sleep activity and location affecting dogs’ sleep variables previously observed (e.g. more REM sleep in dogs sleeping at home versus at the laboratory)^66^, in our study, had no significant associations and were unlikely affected by ADHD traits in dogs. Therefore, although all dogs were assessed under the same condition and our findings of within-individual relations between age and sleep parameters may not be affected by these factors, it is recommended in future studies the assessment of home and/or nighttime recordings to evaluate these relationships under different conditions.

Also, it is possible that our results related to sleep EEG variables were affected by a first/second-night effect at least to a certain extent (dogs^50^; humans^71^). Further, dogs’ sleep habits (frequency of sleeping away from home) also affect their sleep pattern, especially on the first sleep occasion in a new environment^50^. In the current sample, we had no information on most dogs’ sleep habits, as their sleep assessment had been conducted before the paper on dogs was published^50^. Thus, in future dog sleep studies, it is suggested to consider dogs’ sleep habits in order to address this effect in dogs.

Finally, objective behavioral evaluations of executive functions could provide complementary information about some dimensions of ADHD and their relationship with sleep, such as performance observed in a Go/No-Go paradigm in dogs^38,72^. Many studies have indicated that disagreements on ADHD diagnostic and symptoms of the subtypes might be one of the fundamental biases in analyses trying to establish objective measures of sleep disturbances in ADHD patients^25,26^. Thus, behavioral tests and an improved version of dog ADHD questionnaire (e.g.^43^) can be helpful in this direction.

## Conclusion

In this large-scale non-invasive sleep study on owner-rated ADHD questionnaire and sleep EEG parameters, we found that some objective indices of sleep are associated with higher ADHD scores in dogs. This suggests that similar neural features might underlie dogs’ and humans’ natural variation of hyperactivity/impulsivity.

## Supplementary Information

Supplementary Information.

## Data Availability

The datasets used and/or analyzed during the current study are available from the corresponding author on reasonable request.
