# Supplementary material for: Owner-rated hyperactivity/impulsivity is associated with sleep efficiency in family dogs: a non-invasive EEG study
Source: Sci Rep. 2023 Jan 23;13:1291. doi: 10.1038/s41598-023-28263-2 (PMC9870861; doi:10.1038/s41598-023-28263-2)
Supplement: Supplementary file 1 — Supplementary Information. [file 41598_2023_28263_MOESM1_ESM.docx]

# SUPPLEMENTARY INFORMATION to:

**Owner-rated hyperactivity/impulsivity is associated with sleep efficiency in family dogs. A non-invasive EEG study**

Cecília Carreiro ^1,2,*†^, Vivien Reicher ^1,3*^, Anna Kis ^2,4^ and Márta Gácsi ^2,3^

^1^ Doctoral School of Biology, Institute of Biology, ELTE Eötvös Loránd University, Budapest, Hungary

^2^ Department of Ethology, Institute of Biology, ELTE Eötvös Loránd University, Budapest, Hungary

^3^ MTA-ELTE Comparative Ethology Research Group, Budapest, Hungary

^4^ Institute of Cognitive Neuroscience and Psychology, Research Centre for Natural Sciences, Budapest, Hungary

^*^These two authors contributed equally to this work.

^†^Correspondence: ceciliacarreiro@gmail.com

**Questionnaire scoring**

Due to the transition from paper to the online ADHD questionnaire, a fault happened related to the answer options. For the first 34 dogs, the questionnaire version (here referred as A) had a four-level rating scale: 0A (never), 1A (sometimes), 2A (often) and 3A (very often). For the last 52 dogs, the questionnaire (here referred as B) had a five-level scale: 1B (strongly disagree), 2B (disagree), 3B (neither agree nor disagree), 4B (agree) and 5B (strongly agree). In order to use all the answers as a single database, we merged the scales. Since five-level Likert scales have better precision and distribution than four-level scales, we chose to modify the less accurate scale (0A-3A), considering the distribution and the distance of its intermediate values (1A, 2A) related to its extreme values (0A, 3A). To control for distortions, the extreme values (0A, 3A; 1B, 5B) were fixed (0A=1B; 3A=5B) to make a combined scale (here referred as C: 1C-5C). Given the fact that four-level scales do not have a neutral answer (e.g. 3B. neither agree nor disagree), we did not force this value in the results of the dogs with the four-level scale (A), but the neutral score was kept (3B=3C). Then, its intermediate values (1A, 2A) were combined with the respective intermediate values of the five-level scale (1A=2B; 2A=4B). Finally, the original answers on scale (A) were substituted by the corresponding values of the combined scale (C) and the answers on scale (B) were kept the same, resulting in one single rating system (1-5) for all dogs. Further statistical analyses were performed with the ADHD values to check the validity of our merging process. The scores of the two scales after merging (**Table S1**) were better distributed (M*_merged_* ± SD: 24.84 ± 6.13, Shapiro-Wilk: *p* = 0.145) than before merging (M*_not_merged_* ± SD: 19.31 ± 8.91, Shapiro-Wilk: *p* = 0.031) and had the same direction with age (**Figure S1**).

**Table S1.** Process of merging the two different level scales (A and B) into one valid single rating system (C).

| **Values/Options** | **Four-level scale (A)** | **Five-level scale (B)** | **Combined scale (C)** |
| --- | --- | --- | --- |
| extreme | 0A. never | 1B. strongly disagree | 1C (0A;1B) |
| intermediate | 1A. sometimes | 2B. disagree | 2C (1A;2B) |
| neutral | - | 3B. neither agree nor disagree | 3C (3B) |
| intermediate | 2A. often | 4B. agree | 4C (2A;4B) |
| extreme | 3A. very often | 5B. strongly agree | 5C (3A;5B) |


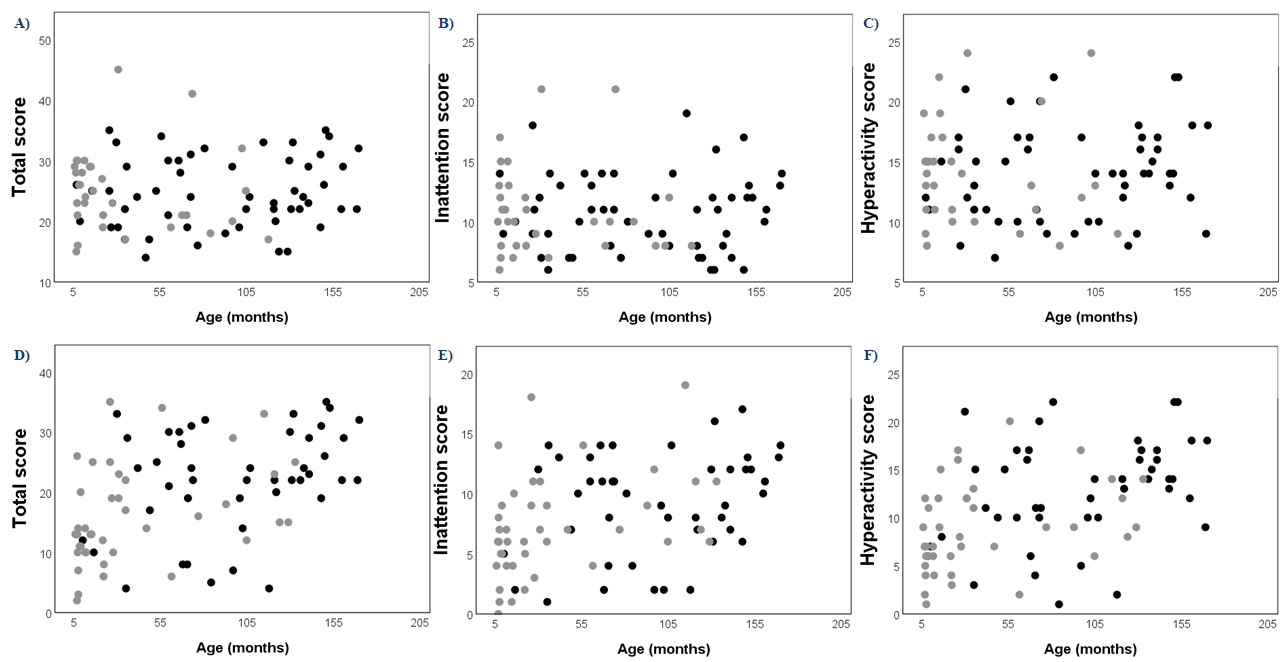


**Figure S1.** Distribution of the three ADHD factors (total, inattention and hyperactivity/impulsivity scores) of the merged scales (A-C) and the different scales (D-F). After merging, both scales became well distributed and had the same direction with age. Black: five-point scale. Gray: four-point scale.

**EEG calibration**

To correct for differences in EEG filter characteristics across recording devices, a calibration process was implemented on the devices described on the section Methods - Electroencephalography: (1) NuAmps amplifier and (2) SAM 25R EEG System. Specifically, a waveform generator at the Fz electrode input of both devices was used to apply 40 and 355 μV amplitude sinusoid signals at various amplitudes (0.05 Hz, every 0.1 Hz between 0.1–2 Hz, every 1 Hz between 2–20 Hz, every 10 Hz between 10 Hz-100 Hz). The amplitude reduction rate for each recording system was determined by calculating the proportion of digital (measured) and analog (generated) amplitudes of sinusoid signals. Next, amplitude reduction rates were calculated for each device and EEG spectrum amplitudes were corrected by dividing such calculated values by the obtained amplitude reduction rate for the recording system.
